# Nanoplastic detection and quantification in high-altitude glacial snow samples from Italy, Switzerland and France

**DOI:** 10.1038/s41598-026-63263-y

**Published:** 2026-08-10

**Authors:** Milena Latz, Alasdair J. Gill, Nikolaos Evangeliou, Sabine Eckhardt, Robin Milner, Thorsten Reemtsma, Dušan Materić

**Affiliations:** 1 Department of Environmental Analytical Chemistry, Helmholtz Centre for Environmental Research (UFZ), Leipzig, Germany; 2 Department for Atmospheric & Climate Research (ATMOS), Stiftelsen NILU (former Norwegian Institute for Air Research), Kjeller, 2007 Norway; 3 High Level Route, https://www.high-level-route.com/, London, United Kingdom

**Keywords:** Climate sciences, Environmental sciences

## Abstract

Plastic pollution is an ever-growing concern around the globe, with current research portraying the presence of contaminants even in previously pristine regions. Especially small particles, micro- and nanoplastics, can be dispersed over long stretches by atmospheric transport, reaching even the most remote areas of the planet. This study aims to portray current levels of nanoplastic contamination (< 1 μm) by analyzing snow samples from high-altitude glaciers in the European Alps (2300–3800 m) via TD-PTR-MS. We were able to detect six common plastic pollutants, with PE and PP contributing over 60% of the total mass concentration measured across all sites. On average, nanoplastic concentrations of 85 ng mL^− 1^ (range: 3.6 ± 12 ng mL^− 1^ – 470 ± 76 ng mL^− 1^) were detected at sampled glacier surfaces. Access to these remote regions was enabled through a citizen-science initiative, involving trained mountaineers for sample collection. The results were applied to atmospheric modelling, highlighting possible point sources of contamination. While agriculture and local plastic factories were revealed as potential sources of nanoplastics, spatial and temporal limitations complicated comparison inside the sample cluster.

**Supplementary Information:**

The online version contains supplementary material available at 10.1038/s41598-026-63263-y.

## Introduction

In 2022, plastic production in Europe alone accounted for a mass of 58.8 Mt in total. From this, over 80% of the products are still sourced from fossil-based plastic, with less than 20% being made from recycled materials and thus being considered circular products^1^. According to the OECD, global plastic production reached 435 Mt in 2020 and is forecast to rise by up to 70% by 2040^2^. Given that only 60% (32.2 Mt) of the total plastic consumed in Europe is being collected and treated post-consumer, mismanaged plastic waste enters our environment on a large scale^1^. Globally, 81 Mt of plastic waste was mismanaged in 2020, with a projected increase of nearly 50% by 2040^2, 3^.

With global plastic production constantly rising, environmental pollution through plastic waste will continue to be a problem of increasing seriousness^4^. While large plastic debris is directly visible to the human eye, pollution by smaller particles has been largely overlooked and underestimated. Once this became evident, multiple fields of research have increased their focus on particles < 5 mm, including microplastics (MPs, 5 mm–1 μm) and nanoplastics (NPs, < 1 μm)^5^. Especially in the aquatic^6, 7^ and terrestrial^8^ecosystems, small plastic particles have been studied more thoroughly, revealing their acute risk to biomes and biota^9, 10^. In recent years, the influence of a third domain - the atmospheric system - has surfaced, uncovering the complex biogeochemical cycle MPs and NPs are subjected to in the global environment.

To obtain a broader picture of both MP and NP pollution in the environment, the role of atmospheric pathways has subsequently been studied in more depth^11, 12^. Generally, pollutants can enter or exit the atmospheric system through suspension and deposition, respectively. Once in the atmosphere, plastic particles can travel long distances, being dispersed by the wind^13^. In particular, smaller particles and fibers with a lower weight and density remain in the atmosphere for longer periods and are transported over longer distances, reaching even the most remote places^10^. This is evidenced by research that has detected plastic contamination in secluded regions of the Antarctic, Svalbard, and Siberia^14, 32, 57, 58^. With wider reporting of these results, particles < 1 μm (nanoplastics) have thus gained more attention with the general public. However, their detection remains complex, mostly limited by their small size. Common analytical techniques that have successfully been implemented are described elsewhere in detail^15, 16^.

Sampling of airborne micro-and nanoplastics can generally be achieved in two ways: active- and passive sampling. During active sampling, particles are deposited on a filter membrane, by passing the sampled air through a cascade impactor device. Passive sampling simply collects all particulate matter from wet and/or dry deposition in an open sampling vessel. Generally, wet deposition is collected during any form of precipitation event, while dry deposition occurs through the continuous settling process of particles. An alternative sampling approach of atmospheric particles is the collection of deposited surface snow. Since snow acts as a scavenger for various contaminants, surface snow samples are the optimal environmental monitor for NPs^17^. Similar to most passive sampling approaches, surface snow samples are classified as bulk sampling, combining both wet and dry deposition. During a snowing event, particles suspended in the air are bound to the snowflakes, either upon their formation in the clouds or during their way to the ground. Consequently, freshly fallen snow typically only contains particles from the wet deposition event itself. Dry deposition only plays a significant role in surface snow that has already been exposed to air for a longer period^17^.

Current evidence is still insufficient to fully evaluate the relationship between human health an NPs accumulation, highlighting the need for further research and comprehensive data on current contamination levels in all parts of the environment. To achieve this, the presented research focuses on the detection of NPs in glacial snow samples from remote regions in the European Alps. This project was realized in collaboration with the High-Level Route (HLR) mountaineering expedition^18^. Snow samples were collected by mountaineers specially trained in NPs sampling, enabling access to otherwise remote and pristine regions.

## Results

### Surface snow samples

**Fig. 1:**
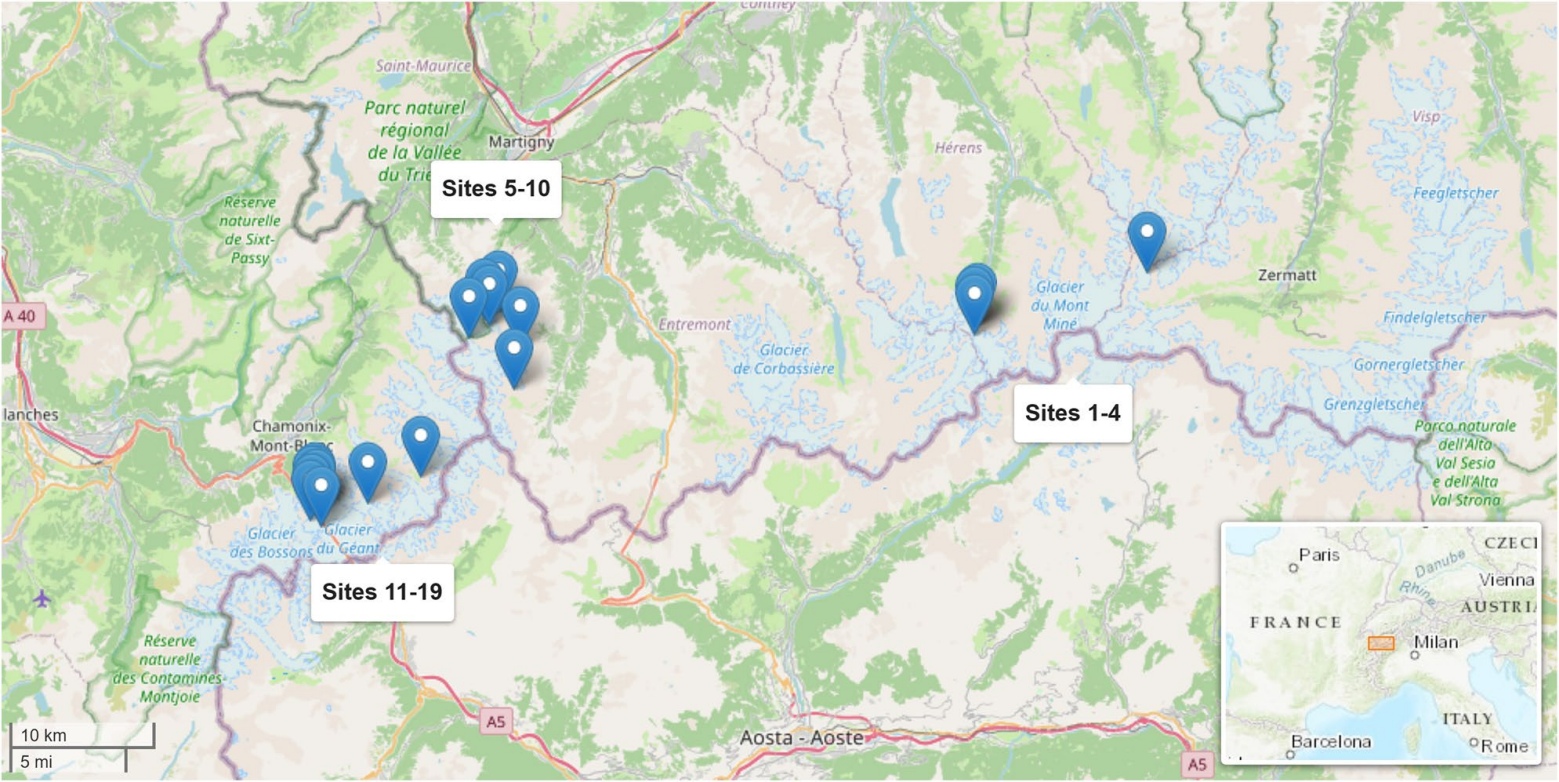
Sampling sites visited during the High Level Route expedition in 2022. Collected samples consisted of surface snow from glaciers in the Alps. The overview map shows the location in the European context. A detailed description of each sampling site can be found in the Supplementary Table S1. Created in R using the leaflet package.

During the presented work, samples from 19 sites (see Fig. 1) were analyzed via Thermal Desorption Proton Transfer Reaction Mass Spectrometry (TD-PTR-MS). This sensitive analytical method employs fast, qualitative and semi-quantitative analysis specifically designed for the detection of both micro- and nanoplastics. Here, we focused solely on the detection of nanoplastics < 1 μm, including six common polymer types: polyethylene - PE, polypropylene - PP, polystyrene - PS, polyvinylchloride - PVC, polyethylene terephthalate - PET, and tire wear particles - TWP.

Selected sampling sites were located at high-altitude levels above 2300 m, with the highest sampling point located at 3801 m (Site 19). As the expedition took place during the summer months of 2022, temperatures ranged from − 1 °C up to 20 °C on days of sampling. Detailed information on each sampling location can be found in the Supplementary Table S1, respectively.

**Fig. 2:**
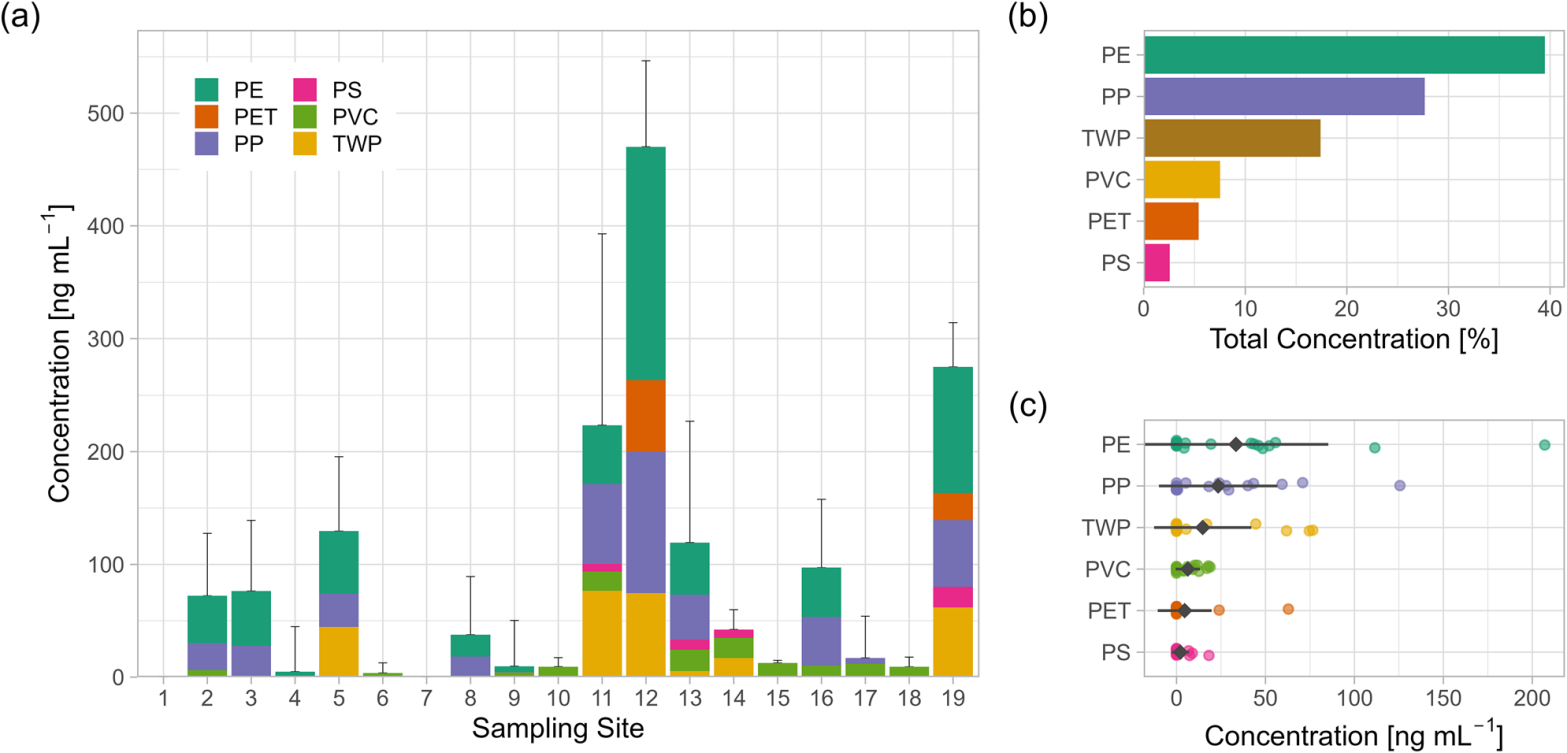
Total detected nanoplastic (< 1 μm) concentrations of six different polymer types (polyethylene (PE), polypropylene (PP), polystyrene (PS), polyvinylchloride (PVC), polyethylene terephthalate (PET), and tire wear particles (TWP)) at 19 sampling sites. With (**a**) total mass concentrations at each sampling site in ng mL^1^(error bars represent the standard deviation of measured triplicates), (**b**) total percentage of polymer types found over all sites, and (**c**) scatter plot of total mass concentrations and mean concentrations (black) in ng mL^-^^1^ over all sites.

Across the 19 sampling sites, all six polymers were successfully detected. Figure 2 portrays a comprehensive overview of measured mass concentrations. In total, nanoplastic particles < 1 μm of varying composition were detected at all sampling sites, except at sites 1 and 7. Mean mass concentrations at sites showing nanoplastic contamination ranged from 3.6 ± 12–470 ± 76 ng mL^− 1^. Overall, highest nanoplastic mass concentrations were measured at site 12 (470 ± 76 ng mL^− 1^), followed by sites 19 (275 ± 40 ng mL^− 1^) and 11 (223 ± 128 ng mL^− 1^). A possible explanation for high levels at sites 12 and 19 could be their similar exposed location at a mountain ridge and top of the glacier. Sites 6, 4 and 18 however showed the lowest overall nanoplastic concentrations, ranging from 3.6 ± 12 ng mL^− 1^ (PVC only), to 4.8 ± 30 ng mL^− 1^ (PE and PP), and 9.1 ± 14 ng mL^− 1^ (PVC only), respectively.

PE and PP were found to be the most abundant contributors towards nanoplastic particles in this study, making up 39% and 28% of the total mass concentration measured. Both polymers were detected at 12 and 14 of all 19 sites, respectively. Interestingly, all 12 samples showing PE concentrations also showed presence of PP.

With 17%, TWPs were the third most abundant contaminants detected at only six sites in total. Comparably, PVC accounted for only 8% of the total mass concentration detected, while being represented at a total of 11 out of 19 sites. While TWPs were present at higher concentrations, PVC showed lower overall concentrations, being represented at a larger number of sites (see Fig. 2, c)).

The lowest mass concentrations were measured for PET and PS, accounting for only 5% and 3% of the total nanoplastics found. While PET showed a higher overall mass concentration compared to PS, PET was only detected in 2 samples (sites 12 and 19). While PS was detected in 4 samples (sites 11, 13, 14, and 19), generally at lower concentrations.

**Fig. 3:**
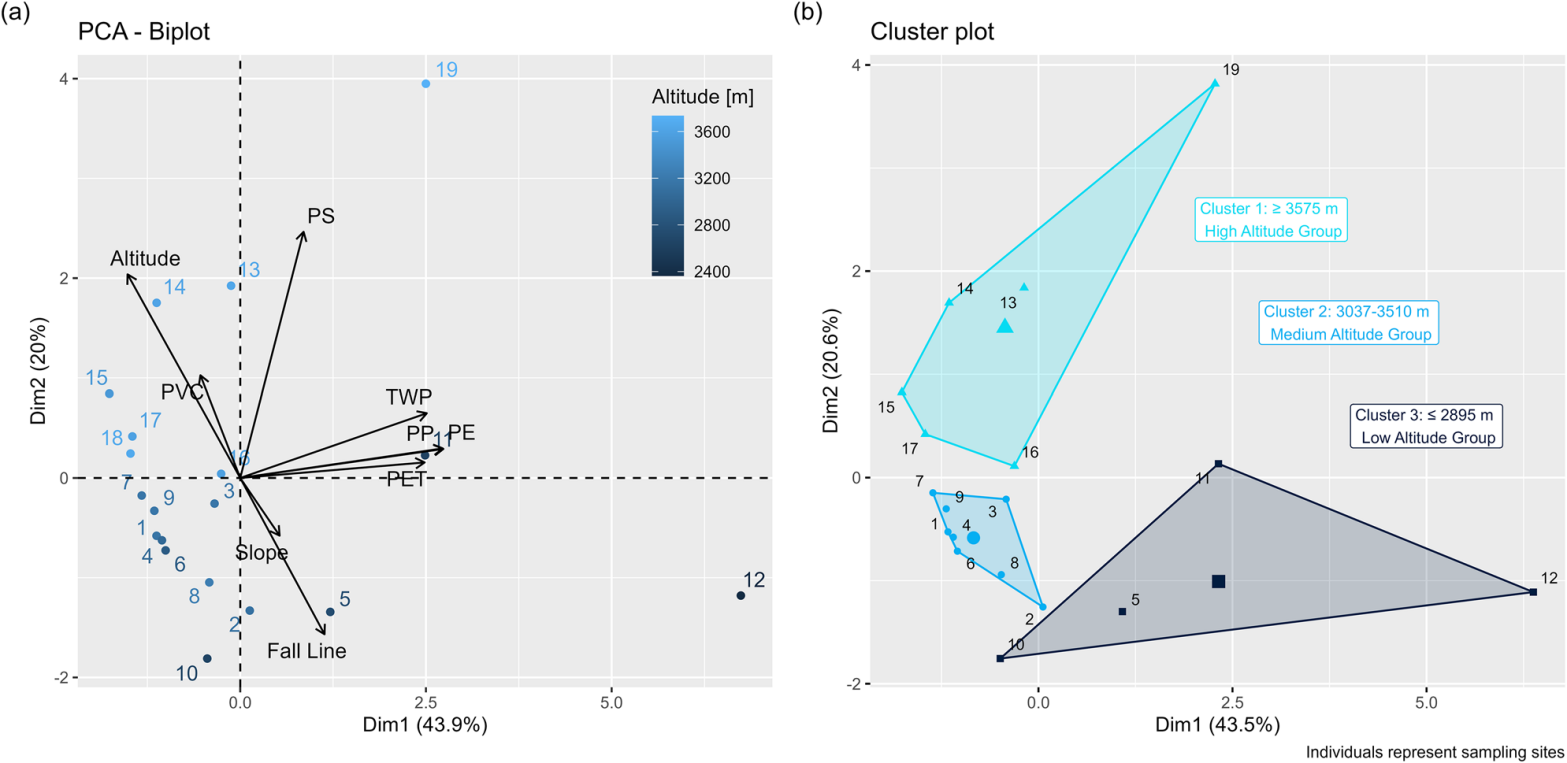
Multivariate Analysis of the presented dataset. With (**a**) portraying Principal Component Analysis (PCA) based on 9 continuous variables (six polymer concentrations (PE, PP, PS, PVC, PET, TWP) and site-specific parameters (fall line, slop, altitude)) and (**b**) portraying K-means Cluster Plot (K = 3): Formation of three distinct clusters based on the site’s altitude: Cluster 1 – high altitude sites (≥ 3575 m), Cluster 2 – medium altitude sites (3037–3510 m), Cluster 3 – low altitude sites (≤ 2895 m). Individuals represent sampling sites. Rows including missing data were omitted from the cluster analysis.

Subsequent statistical analysis revealed no significant linear correlation (*p* < 0.05) between total detected concentration and other site-specific parameters (slope, fall line, altitude). Similarly, we could not detect any clear trend across the samples through Principal Component Analysis (PCA) alone (Fig. 3a). PC1 and PC2 explained 43.9% and 20.0% of the total variance, respectively. Variables from PE, PET, PP, and TWP concentrations exhibited strong positive loadings in the first dimension (PC1), whereas PVC and PS concentration variables were strongly associated with the second dimension (PC2). Slope and fall line were oriented in a similar direction, suggesting a positive correlation between the two variables. In contrast, altitude variables were oriented in the opposite direction. Individuals (sampling sites) presented in the PCA were mainly grouped in the bottom left quadrant of the plot, except for site 19 (top right) and site 12 (bottom right). Interestingly, a second exploratory PCA analysis including only site-specific parameters revealed a different orientation for each of the three variables and an increased scattering of the individuals across the plot (see Supplementary Figure S2c).

For subsequent K-means Clustering, K = 3 was specified as an optimum using the silhouette method and the elbow method. Due to missing data, site 18 was excluded from this analysis. Figure 3b reveals a distinct grouping of individuals into three clusters. Further analysis of the frequencies in each cluster shows grouping based on the sampling site’s altitude. We found no clear grouping for the other variables. Detailed information on all statistical analysis can be found in the Supplementary Figure S1-S3 and in the Supplementary Table S1, respectively.

### Upscaling estimation

To extend the sampling results generated to a larger context, the total amount of NPs detected was upscaled to the glacial area of the European Alps. According to Paul et al.^19^, a total glacial area of 1806 ± 60 km^2^ can be assumed in 2016. By including an accumulation-area ratio (AAR) of 0.58^20^±0.19 as well as a shrinkage factor of 1.2% a^− 1^, the total accumulation zone in the sampled year 2022 was calculated to 972 ± 322 km^2^. Based on our sampling method (see Supplementary Methods), the jars’ base area can be assumed as the total surface area sampled. From this, we derived a transformation factor of 0.4 cm^2^ mL^− 1^, enabling conversion from sample volume to sampled surface. Correspondingly, detected NPs mass concentration values of 85 ng mL^− 1^ on average translate to 2.2 kg per km^2^ of sampled surface. A systematic error of ± 30% for the analytical method, an AAR uncertainty of ± 33% (added to account for fast changing accumulation zones in recent years), as well as an experimental uncertainty between sites of ± 33%, and a sampling error of ± 24% were assumed. Error propagation subsequently revealed a total uncertainty of ± 61%, assumed for the final upscaled value. In conclusion, a total nanoplastic concentration of 2.1 ± 1.3 t can be assumed for the total glacial area of the European Alps. A detailed calculation and error propagation can be found in the Supplementary Equations S1-S11.

Given the substantial total error of over ± 60% and the presence of multiple uncertainties directly affecting the result, this value should be regarded only as a broad reference estimate. First of all, the variability of detected concentrations between all sampling sites is quite high. Furthermore, only a small fraction (3 × 1 mL) of the total sample (~ 40 mL) was analyzed throughout this study. While a low sample volume is generally advantageous to minimize sample loss, the overall error increases with smaller fractions taken. It is therefore not possible to reflect the quality and quantity of nanoplastics on a large, environmental scale with complete certainty through simple extrapolation of the generated results as assumed here^21^.

### Potential origin of the transported plastics

**Fig. 4:**
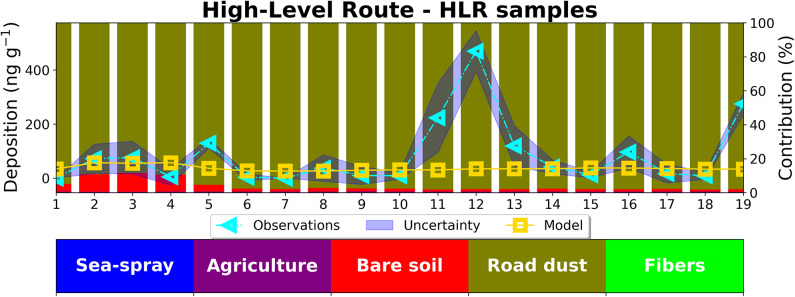
Modelled source contribution of atmospheric plastics to sampling points during the HLR campaign. Bars correspond to the right y-axis. Observations (cyan triangles) against modelled deposition (yellow rectangles) are also plotted.

Modelled deposition densities of atmospheric plastics along with the observations for the HLR campaign can be seen in Fig. 4. While FLEXPART coupled with the emissions produced reasonable estimates of accumulated deposition, the model failed to capture the finer-scale variation present in the observations, particularly for samples 5, 11–13, 16, and 19. This mismatch occurs, because the spatial resolution of the model is coarser than the distance between the sampling sites and, in turn, transport cannot be resolved by the model. Even though the sampling points during the HLR campaign were located in very close proximity to one another (10–50 km apart), observed NPs show quite different chemical compositions, due to differences in topography, leading to differences in wind and precipitation patterns, which are not resolved by the underlying input meteorological fields used (0.5° × 0.5°). An additional reason for the poor performance of the model is that local circulation patterns over complex mountainous orography cannot be captured by any available meteorological field independent on the resolution. Smoothed model orography can lead to substantial differences between the real station altitude and the model surface height, complicating the representation of whether the receptor samples boundary layer or free tropospheric air. Such representativeness issues, together with uncertainties in boundary-layer structure and turbulence in complex terrains, can bias simulated source-receptor sensitivities and the timing and magnitude of regional contributions^22, 23^.

**Fig. 5:**
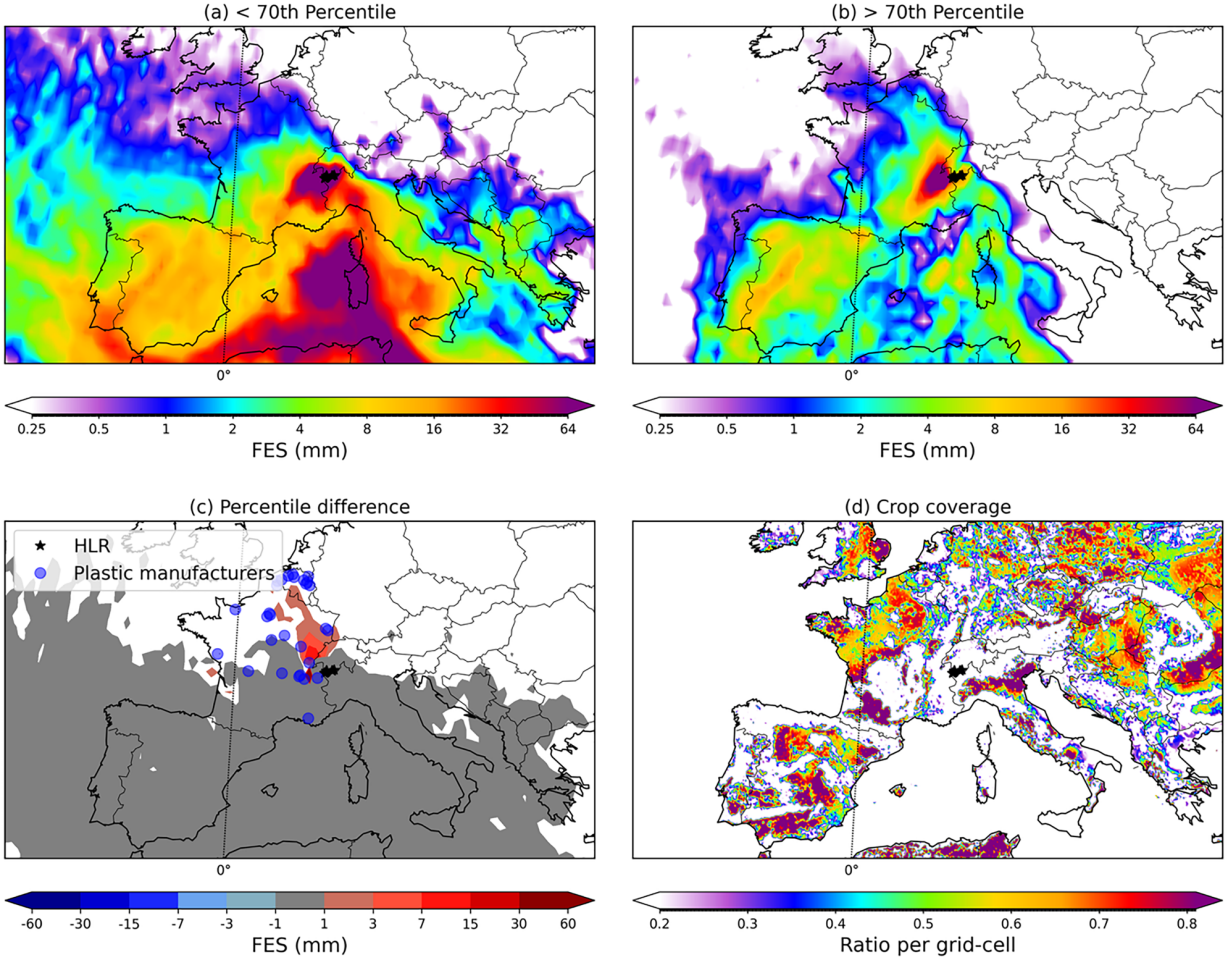
Average footprint emissions sensitivities (FES) for those samples (**a**) under the 70th percentile (the majority of the samples had values under 100 ng g-1), (**b**) those that exceeded the 70th percentile (samples 5, 11–13, 16, and 19), and (**c**) their difference (blue circles indicate the location of the main plastic manufacturers and distributors in France and Belgium). (**d**) Croplands and pasture coverage are plotted as a potential source of plastic pollution due to the extensive use of agricultural nets. Black stars in the borders of France, Italy and Switzerland are the sampling points of HLR.

A more detailed analysis of the modelled long-range transport is depicted in Fig. 5. The figure shows the average footprint emissions sensitivities (FES) corresponding to the samples that are below the 70th percentile or 98 ng g⁻¹ (Fig. 5a), the average FES for the samples above the 70th percentile (Fig. 5b), their difference (Fig. 5c), and the gridded coverage of cropland and pasture (Fig. 5d) from Ramankutty et al.^24^. The structure of FES for the low deposition samples is very similar to that of the high deposition samples, although the intensity is clearly different. This similarity implies that in both cases the predominant origin of plastics arriving at the receptor points of HLR lies in southern source regions (Fig. 5a and b). However, the fact that the two FES show large differences in measured deposition intensity suggests that North Africa or the Mediterranean Sea cannot be the dominant sources in this case. Instead, the variability must be explained by closer or more episodic sources.

To further investigate whether the mismatch between modelled and measured deposition (Fig. 4) arises from missing sources in the emission inventory or from the influence of meteorological variability, we compute the difference between the high and low deposition FES (Fig. 5c). This differential analysis can be viewed as an air-quality assessment tool, highlighting episodic high-deposition events against a more stable background signal. If the high-deposition FES is much stronger than the low one, it indicates that receptor sites are occasionally affected by distinct high-pollution episodes. Conversely, if the difference is small, it would imply that the observed concentrations are largely governed by background levels with little event-driven variability. In our case, Fig. 5c clearly shows that the difference between high and low deposition FES is attributable to a relatively confined region covering eastern and northern France, extending into Belgium.

For these regions, we also plotted the locations of major plastic manufacturing and distribution companies. The spatial coincidence is striking: many of these companies are concentrated in central and eastern France, as well as in Belgium, precisely in the areas where the FES difference signal is strongest. This spatial overlap strongly suggests that the current emission inventories employed in the study may not adequately represent certain industrial or commercial sources of plastics. In other words, the observed mismatch between modelled and measured deposition (Fig. 4) is at least partly explained by unconsidered emissions from the plastics production and distribution sector in this region. This finding emphasizes the importance of refining emission inventories with improved bottom-up data, especially in industrialized regions, to better constrain models like FLEXPART.

 Fig. 5 d shows cropland and pasture coverage across the European domain. Although agricultural activity did not contribute at all (see Fig. 4), the presence of extensive agricultural fields in central and eastern France, as well as in Belgium, coincides with the region where the FES difference is strongest. Whether this is a missing source of atmospheric plastics in Evangeliou et al.^25^ requires further research. 

The sampling times were highly clustered within a short two-week period, during summer 2022, a season typically characterized by relatively few precipitation events in the region. Therefore, the modelled deposition fluxes are very similar, as they were calculated for the same time and also for similar locations. Consequently, snow accumulation began at very similar dates across all sampling sites, effectively synchronizing the deposition signal across the network. The source regions for deposition change on synoptic scales within few days. In FLEXPART, the start of the footprint for deposited mass is the time where precipitation started and end time is the time that snow accumulated until its mass reached the exact collected amount of snow. In order to have a best estimation of source regions, several short periods of sampling would be optimal, which would require collections of small snow samples. This would enable us to resolve different source regions contributing to the different long-range transport events, which are now averaged over a longer sampling period. In practice, the FES therefore lacked strong contrasts that could be used to disentangle contributions from distant source regions. However larger regions of MP sources can be identified and provide valuable insights into the origins of atmospheric plastics during the campaign. The largest source sector feeding plastics into the Alps during the HLR sampling campaign was calculated to be road dust, contributing more than 80% of the total deposition (Fig. 4). This highlights the dominant role of traffic-related microplastic emissions in shaping deposition fluxes, even in remote alpine environments. A smaller but notable contribution came from the resuspension of bare soils, which likely reflects the entrainment of degraded plastic fragments previously deposited on land surfaces. All other source sectors, including urban waste handling, industrial emissions, and long-range transport from marine sources, were negligible during the campaign. These results reinforce the emerging consensus that secondary re-emission processes (road dust, soil resuspension) are among the most important drivers of atmospheric microplastic burdens, especially in continental interior regions such as the Alps.

## Discussion

According to PlasticsEurope 2022, PE and PP are the plastic types most commonly produced in Europe, making up 15.4% (PP) and 22.1% (all PE) of the market. Their main application area is packaging materials[1]. In comparison, PET is commonly utilized to produce plastic bottles, whereas PS is employed in its foamed form (Styrofoam) as packaging material^2, 26^, In 2020, each polymer type made up 5% of the total plastic produced in Europe^1^. Worldwide, packaging materials are the most common area of application for plastic materials, followed by transportation and textile production^2^. Due to their ability to float on water, lower density plastics such as PE and PP are more prone to fragment into smaller MP or NP particles through their exposure to sunlight in the oceans^2, 27^, Apart from their higher production rate, an increased degradation of PE and PP could also account for the large contribution to the total measured mass concentration observed in this study.

These results highlight the large plastic problem we are facing. While measures have been taken in Europe, decreasing the total plastic production by 5.6% since 2018 and slowly shifting from fossil-based resources to circular plastics (+ 30% since 2018), this does not solve the problem^1^. Especially small particles such as NPs are difficult to remove from the environment and will only accumulate over time due to their low degradation rates^28^. Even if plastic production will continue to decrease, mishandled plastic waste that has already entered the ecosystems must be handled. For this, further research has to be conducted on removal processes. Furthermore, standard operating procedures for the analysis of MPs and NPs in the environment have to be established in order to allow for comparable results. With this, the severity of plastic pollution in the environment can be highlighted, leading to the implementation of regulations.

The practice of collecting snow samples as environmental monitors for airborne pollution in remote regions is a method well known in literature already since the 1980s^29^. Due to size dependent limitations of most analytical methods, previous research mainly focused on the detection of MPs > 100 µm^17, 30, 31^ or on smaller microplastics > 1 µm^32–34^. With the rise of novel analytical techniques, studies implementing the analysis of nanoplastics into their research have surfaced in recent years (see Table 1)^14, 35–39^.

**Table 1:** Brief literature overview on selected methods for the detection of nanoplastics in snow samples.

References	Method	Plastic types	Sample aliquots	Size fraction	Detection limit	Total quantity
This work	TD-PTR-MS	PE, PP, PS, PVC, PET, TWP	1 mL	< 1 μm	N.A.	85 ng mL^− 1^
Belontz et al.^35^ (06/25)	AFM-IR, O-PTIR	P(3HB-*co*−4HB)	80 µL	All	10 nm (AFM-IR) 500 nm (O-PTIR)	150 ng mL^− 1^
Wang et al.^36^ (03/25)	HoLDI-ToF-MS	PE, PEG, PDMS, PS, PET	500 mL	> 0.1 μm	3.0 mg mL^− 1^ (PE) 5.0 × 10^− 4^ mg mL^− 1^ (PEG) 0.12 mg mL^− 1^ (PDMS) 2.5 mg mL^− 1^ (PS) 8 mg mL^− 1^ (PET)	0.06 mg L− 1 (60 ng mL^− 1)^
Jurkschat et al.^14^ (01/25)	TD-PTR-MS	PE, PP, PS, PVC, PET, TWP	1 mL	< 1 μm	0.7–1.2 ng mL^− 1^ (PET) 2.4 ng mL^− 1^ (PS)	2–80 ng mL^− 1^
Materić et al.^37^ (11/21)	TD-PTR-MS	PE, PP, PS, PET, TWP	1.5 mL	< 0.2 μm	N.A.	46.5 ng mL^− 1^
Wang et al.^38^ (05/21)	SALDI-MS, NALDI-MS	PEG, PE	1 µL	All	80 pg (PEG); 100 pg (PE) 5 pg (PEG); 100 pg (PE)	121.9 ng mL^− 1^
Materić et al.^39^ (01/20)	TD-PTR-MS	HDPE, LDPE, LLDPE, PPC, PP, PS, PET, PVC	1 mL	< 0.2 μm	0.34 ng (PS)	22.9–23.5 ng mL^− 1^

Detected concentration values of NPs < 1 μm are within the same order of magnitude, compared to previous results from studies in literature. Nonetheless, it remains difficult to directly compare generated results with earlier studies, as the analyzed size fraction as well as the analytical method used often differs between studies. Similar conditions (analytical method, particle size) can be assumed for the study conducted by Jurkschat and coworkers^14^. In both cases, glacial snow was collected during the summer months in the European Alps, analyzing all particles < 1 μm via TD-PTR-MS. Throughout their study, Jurkschat et al.^14^ detected NP concentrations in the range of 2–80 ng mL^− 1^, being in a comparable range to NP concentrations detected in this study (85 ng mL^− 1^ on average). Interestingly, Jurkschat et al.^14^ have found high concentrations of TWP (41%) and PS (28%) in the analyzed samples, while both polymer types only account for a smaller fraction in this study. Coherently, both studies detected a high content of PE in the sample (12%^14^/39% (this study)).

A comparable study by Materić and coworkers revealed average mass concentrations of 46.5 ng mL^− 1^for nanoplastic particles < 0.2 μm in surface snow samples from the Austrian Alps^37^. The lower concentrations observed in this case can be a possible result of varying reasons: (1) the smaller particle size fraction analyzed, (2) the higher number of observed snowing events throughout the sampling campaign (20 out of 41 days), leading to additional dilution effects. With fresh surface snow occurring every other day, this suggests a large fraction of the sample accounting for wet deposition. (3) the sampling period (Feb-March): lower deposition rates can be expected from October to February, due to the varying efficiency of upward transport in the atmosphere^36^. While similar mass concentrations could be measured in total, all three studies only represent a short snapshot of a specific time frame, combining various contamination sources. It is to note that all results being measured via TD-PTR-MS have to be seen as semi-quantitative, and an error rate of ± 30% should always be kept in mind.

The interpretation of the portrayed FLEXPART modelling is consistent with the broader understanding that atmospheric plastic pollution is often dominated by secondary emissions from terrestrial sources such as industrial sites, road dust, and bare soils rather than from long-range marine transport^17, 40^, The strong regional signal in France and Belgium highlights the need for more detailed local inventories and underscores the sensitivity of receptor-based analyses to nearby industrial clusters. Moreover, given the relatively short transport distances involved, small differences in meteorological conditions (e.g., boundary layer height, precipitation events, or frontal passages) may amplify the contrast between high and low deposition samples. This suggests that both improved emission inventories and higher-resolution meteorological inputs are needed to fully reconcile the differences between measurements and model results. Agricultural activities are increasingly recognized as an important source of plastic emissions into the environment, both directly and indirectly^41, 42^, Direct sources include plastic mulching films, widely used to retain soil moisture, control weeds, and improve crop yields. Over time, these degrade into microplastics through mechanical stress, UV radiation, and tillage, with fragments released into soils that can later be resuspended into the atmosphere and transported by wind^43^. Greenhouses and plastic tunnels represent another direct source, where covers and sheeting fragment via weathering, abrasion, or poor disposal practices^44^. Finally, plastic irrigation pipes and fittings can undergo mechanical wear and photodegradation, releasing fragments into soil and air. Indirect sources include fertilizers and soil amendments, particularly biosolids and composts that contain microplastic contaminants^45, 46^, Their application spreads microplastics across agricultural land, from which they may be resuspended into the atmosphere. Another indirect source is plastic-coated fertilizers and pesticides that use polymer coatings which degrade and fragment, adding microplastic particles to the soil–air system^47^. Agricultural machinery, such as tractors and other farm vehicles, also contributes through tire wear particles^48^.

As described previously, collected surface snow acts as an environmental monitor for airborne particulates such as MPs and NPs. Measured polymer concentrations in the samples thus also reflect airborne plastic contamination levels to some extent^29^. While research on airborne MP particles and their influence has increased, linkages between airborne NPs and environmental- or human health effects are still contradicted. It has become evident, that the occurring effects are dependent on the size of airborne plastic particles. Larger particles like MPs provide, especially under harsh external conditions, a favorable habitat for microorganisms (known as the “plastisphere”) in all ecosystems including the atmosphere. Conversely, smaller sized NPs are unsuitable for hosting organisms larger ≥ 1 μm like bacteria, fungi, or microbial cells. Alternatively, their higher surface reactivity facilitates hetero-aggregation with non-plastic particles or smaller contaminants such as viruses, genes, or chemicals^49, 50^, While NPs are therefore less prone to be inhabited by microorganisms, their presence in the atmosphere still aids the distribution of certain diseases or pollutants. Moreover, airborne NPs are indirectly influencing the global climate and weather through cloud interactions. NPs can thereby act as ice nucleating or cloud condensation particle^51, 52^.

Similar to MPs, atmospheric NPs can also be taken up by humans and other mammals through inhalation. Though compared to larger MPs, their smaller size facilitates the uptake deeper into the lower respiratory system. The penetration depth is thereby mainly dependent on the size as well as the density of the inhaled particle^53, 54^, While larger particles < 5 μm can accumulate in the small airways or the alveoli, particles < 1 μm can possibly be transported far deeper into the body. Their ability to penetrate biological membranes e.g. the gut-lung-skin barrier, enables them to even reach into the bloodstream^55^. While recent studies have suggested a linkage between the inhalation of MPs and NPs and possible health risks for humans and mammals^55^,a definite conclusion or linkage requires further research, especially focusing on higher environmental relevance^50^.

In addition to the generated results, the presented study highlights the importance of citizen science projects. Collaborations like the High Level Route Expedition allow for the execution of open science, engaging the broader public in science. With the help of citizen scientists, large and long-term datasets can be implemented as a team, otherwise beyond the scope of one researcher. In our case, citizen science additionally facilitated the collection of samples in remote and possibly dangerous mountaineering areas, further increasing the scope of environmental research^56^. To generate scientific reliable and high-quality results from a project employing citizen science, a standard operating procedure must be established and consistently employed throughout the sampling campaign. Similar to other sampling expeditions, quality control procedures are essential, enabling the tracking of possible trace contaminations to its source. To ensure high-quality data, all mountaineers employed in the sampling process have been extensively trained, clarifying the importance of precise work as well as the dangers of possible cross-contamination. Additional sampling information (e.g. worn clothing by samplers, weather conditions) have been noted across the sampling campaign.

## Conclusion

Looking ahead, the HLR campaign and the results highlighted by this study both underscore the value and the limitations of regional field campaigns for model evaluation. While observations provide critical ground truth, the combination of short sampling duration, closely spaced sites, and relatively coarse model forcing data constrains the ability of transport models like FLEXPART to replicate small-scale variability. Future work could benefit from longer observation periods, inclusion of high-resolution meteorological inputs (e.g., convection-resolving models), and integration of complementary tracers (such as black carbon or mineral dust) to better constrain transport pathways. Nonetheless, the dominance of road dust as a source highlights a clear policy lever for reducing atmospheric plastic pollution through mitigation of tire wear particles and improved dust management practices.

## Materials and methods

### Chemicals and materials

#### Chemicals

Chemsolute HPLC-grade water was purchased from Th. Geyer GmbH & Co. KG, Renningen. Supelco PS standard solution (monodisperse, 0.5 μm particle diameter, No. 95585) was purchased from Merck KGaA, Darmstadt.

#### Instrumentation

Samples were analyzed using the IONICON PTR-TOF 10k (Ionicon Analytik GmbH, Innsbruck, Austria). Subsequent data analysis was performed using the associated PTR-MS Viewer Software, version 3.4.5.18 (Ionicon Analytik GmbH, Innsbruck, Austria).

### Methodology

#### Sampling

Surface snow samples were collected during the HLR citizen science expedition in 2022 on the glacial traverse “haute route/high level route”^18^. This high-altitude mountaineering route ranges from Chamonix to Zermatt in the European Alps. Further information on the sampling sites can be found in Fig. 1 as well as their exact coordinates in the Supplementary Table S1. Prior to the expedition, sample vials were cleaned and baked at 250 °C to minimize contamination.

Mountaineers collaborating on the HLR project were well trained, following a detailed standard operating procedure (SOP) for the sampling process (see Supplementary Methods). The procedure was developed by Jurkschat and coworkers, and thereby explained in detail^14^. In short: At the designated sampling site, the sampler would open a glass jar (50 mL, Th. Geyer GmbH & Co. KG, Renningen) and push it downwards into the surface snow. To collect a field blank (FB), an empty sampling jar was opened and filled with HPLC-grade water, mimicking the sampling process of snow. All vials were closed tightly using a screw cap with PTFE septa (Th. Geyer GmbH & Co. KG, Renningen), wrapped with aluminum foil and stored in a cotton bag. Upon arrival at the research institute, melted snow samples and FBs were stored in the fridge until analysis. Depending on the density of the collected snow, a final volume of 30–40 mL melted snow was collected.

#### Sample preparation

Sample preparation was carried out in a closed hood wearing a cotton lab coat and no gloves. Weekly cleaning protocols inside the laboratory minimized possible contamination from dust particles in the air and on surfaces. Sample vials, liners and screw caps were baked overnight at 250 °C prior to sample preparation. Upon preparation, samples were gently shaken to homogenize precipitates. About 10 mL of the sample was filtered through a 1 μm PTFE syringe filter (CHROMAFIL Xtra, Macherey-Nagel GmbH & Co. KG, Düren) using a 10 mL PP syringe (Th. Geyer GmbH & Co. KG, Renningen). 1 mL triplicates of each sample were subsequently transferred into 10 mL headspace glass vials (VWR International GmbH, Darmstadt) using PP pipette tips (epT.I.P.S Standard, Eppendorf SE, Hamburg). Vials were closed using aluminum screw caps (VWR International GmbH, Darmstadt), including perforated PTFE liners (in-house built). Samples were dried until complete dryness (4 h), using a low-pressure evaporation/sublimation system consisting of a DURAN^®^Glass Desiccator (Schott AG, Mainz, Germany) equipped with an in-house built stainless-steel plate connected to a diaphragm N813 gas pump (KNF DAC GmbH, Hamburg)^59^. Two sets of procedural blanks (PB), one using HPLC-grade water and one using lab air, were prepared in triplicates under the exact same procedure. For blanks in between measurements, clean vials filled with lab air were used.

#### Measurement

Samples were measured using Thermal Desorption Proton-Transfer-Reaction Mass Spectrometry (TD-PTR-MS) as described in previous literature^14, 39^, The initially liquid sample has been evaporated during sample preparation, leaving all particles < 1 μm deposited in the glass vial. Upon analysis, the sample is heated to 350 °C, emitting (semi-) volatile compounds into the gas phase. Gaseous compounds are then introduced into the PTR-MS via headspace analysis. Analytes are ionized via a proton transfer reaction with H_3_O^+^ ions present in the drift tube, allowing for subsequent detection via a Time-of-Flight Mass Spectrometer (ToF-MS).

To achieve this method, we coupled a commercially available PTR-MS (PTR-TOF 10k, Ionicon Analytik GmbH, Innsbruck, Austria) and an in-house built thermal desorption unit, similar to our other work^59^. In the morning, five blanks were measured to prepare the system for sample analysis. Samples were then measured randomized in blocks of three, with one blank in between. PTR-MS system specifications were set to the following values: E/N: 120 Td, Flow_inlet_: 120 mL min^− 1^, T_inlet_: 180 °C, scanning range: *m/z* 0–400. Thermal desorption was achieved by applying a temperature gradient program: 35 °C for 30 s, ramp at 40 °C min^− 1^ to 350 °C, hold for 3 min before cool down (15 min in total).

### Data analysis

Data analysis was performed using the PTR-MS Viewer Software, version 3.4.5.18 (Ionicon Analytik GmbH, Innsbruck, Austria), resulting in concentration values in ppb for each measured spectrum (one per second)^60, 61^, To calculate the measured values in ppb, formula (1) is applied by the software^62^:

1 $$\:\left[mf\right]=\:\frac{1}{kt}*\frac{\left[M{H}^{+}\right]}{{[H}_{3}{O}^{+}]\:}*\frac{\sqrt{{\left(m/z\right)}_{{H}_{3}{O}^{+}}}}{\sqrt{{\left(m/z\right)}_{{MH}^{+}}}}$$

With *mf* being the mole fraction in ppb, *k* being the reaction coefficient, and *t* being the residence time of primary ions in the drift tube.

Subsequent data processing followed a similar workflow as described in previous work^39^. To minimize human error and increase the overall speed, data processing was performed using Python (version 3.12.8) and Visual Studio Code (version 1.97.2). In short: spectra were integrated over a measurement period of 7 min, starting from a TD temperature of 200 °C, creating one averaged spectrum for each sample run covering the time period of interest. Afterwards, triplicate samples were averaged and corrected for the mean blank concentration and mean procedural blank concentrations (from PBs). The limit of detection (LOD) was set to the mean of all blank measurements minus three times the standard deviation (3σ) of the blanks. Subsequent fingerprinting compared the 40 most abundant ions (qualifying ions) during the integrated window with a previously established plastic library including polyethylene - PE, polypropylene - PP, polystyrene - PS, polyvinylchloride - PVC, polyethylene terephthalate– PET and tire wear particles – TWP. To establish this library, plastic particles < 0.3 mm were created in-house and one particle each (multiple particles for TWP) loaded into a clean, empty vial (baked for 8 h at 250 °C). PE and PVC were provided by TCR Plastics, Netherlands. PET, PP, and PS were taken from clean end products (plastic bottle, laboratory tube, and laboratory cold storage material, respectively). TWP consisted of a mix of three commonly used car tire brands in the Netherlands. Further information on the plastic library including the thermograms and most abundant ions of all six polymer types can be found in the Supplementary Table S5 and in previous literature^39^. Final polymer concentrations in the measured samples were calculated using formula (2), considering sample volume and dilution effects:

2 $$\:\left[c\right]=\:\frac{mf*{M}_{m/z}}{{V}_{M}}*F*{t}_{int}$$

With *M*_*m/z*_ being the molar mass of the respective *m/z*, *V*_*M*_ being the molar volume of ideal gas, *F* being the TD flow rate, and *t*_*int*_ being the integration time.

The final concentrations were received by subtracting all concentration values with the mean concentration of the FBs. The workflow code, fingerprinting algorithm, as well as all raw data and mass spectral plastic libraries can be found in the Zenodo Repository. Calculation of the total standard deviations, creation of graphs, and maps was carried out using R (version 4.4.1) and RStudio (version 2024.12.0).

### Quality control and quality assurance

According to Wright et al.^21^, strict quality control measurements have to be followed during the analysis of MPs in order to receive representable, high quality data of measured concentration levels. Similarly, the analysis of NPs requires equally, if not more, rigorous protocols. Throughout this study, we have therefore employed multiple quality control measures, explained in the following.

To minimize contamination risks during the mountaineering expedition, a SOP (see Supplementary Methods) was implemented to ensure that the sampling process followed precise rules^14^. In short, upon arrival at the sampling site, samplers wore new gloves, collecting undisturbed, pristine snow while being positioned standing in a headwind from the sampling site. Collected sample vials were wrapped in aluminum foil and stored in a cotton bag until arrival at the laboratory. Moreover, meteorological conditions, sampler’s name and worn clothing were noted. To represent possible contamination sources in the field, eight FBs have been collected at different sampling sites and days.

Upon sample preparation, PBs representing possible contamination sources in the laboratory environment were produced containing HPLC-grade water and air. Both sets of PBs showed no sign of contamination originating from sample preparation. This not only indicates a clean preparation process, but also demonstrates that neither the small amount of plastic labware (syringes, syringe filters, and pipette tips) nor the HPLC-grade water used represented potential contamination sources in our case. These results are also in line with previous work evaluating contamination sources in MPs research^63^. The collected FBs revealed small signs of contamination for the two polymers PE and PP. Generally, mass concentrations detected were comparably low on average, ranging from 2.2 ± 11 ng mL^− 1^ (PE), to 12 ± 20 ng mL^− 1^ (PP). No contamination was found for other polymer types. To correct samples for field blank contaminations, FB concentrations were averaged and subtracted from concentrations measured for PE and PP in each sample. All data concerning quality control and quality assurance can be found in the Supplementary Table S3 and S4.

To determine the method’s recovery rate, three samples and FBs each were randomly selected. Triplicates were prepared as previously described (see Sample Preparation). Subsequently, each sample was spiked with 100 ng PS standard solution. For this, 1 µL of a 100 ng µL^− 1^ solution was added using a PP syringe tip (epT.I.P.S Standard, Eppendorf SE, Hamburg). Before measurement, all vials where dried at 60 °C for 1 h. Calculated recovery rates ranged from 86 to 118% (102% on average), indicating a high ionization efficiency and recovery rate of the method. For each triplicate, standard deviation ranged from 10 to 18 ng. The measured recovery rate drastically exceeds previously reported rates based on similar methods via TD-PTR-MS (25%^14^, 31%^64^, 15%^39^). A possible explanation is the use of a newly acquired and advanced PTR-MS instrument, offering higher mass resolution and sensitivity compared to models employed in previous studies.

### Atmospheric dispersion modelling and source quantification

The source origin and transport of the deposited plastics at the HLR sampling points were tracked with the Lagrangian particle dispersion model FLEXPART version 11^65^ driven with ERA5 assimilated meteorological analyses^66^(137 vertical levels, hourly temporal resolution and 0.5° x 0.5° spatial). The footprint emission sensitivities (FES) were calculated in backwards time mode, using a feature that reconstructs separately wet and dry deposition at the receptor for backward simulations^67^. Wet deposition of microplastics was reconstructed after releasing computational particles at the sampling site (receptor) at altitudes of 0–20 km above sea level during precipitation events. Wet removal was calculated using different scavenging coefficients for in-cloud and below-cloud scavenging. For dry deposition, particles were released at 0–30 m at the same receptor point, as this shallow layer is equal to the height of the layer in which, in forward mode, particles are subject to dry deposition. All released particles represent a unity deposition amount, which was converted immediately (i.e. upon release of a particle) to atmospheric concentrations using the deposition intensity as characterized by either the dry deposition velocity or wet scavenging rate (in-cloud and below-cloud scavenging)^67, 68^. This gives the sensitivities between emissions and deposition amounts using 30-day backward tracking assuming long atmospheric lifetime of NPs.

FLEXPARTv11 is suitable to model MPs and NPs considering shape corrections during gravitational settling of shapes other than spheres^69^. Accordingly, we have assumed that the modelled MPs/NPs are fragments < 10 μm. Except for gravitational settling and dry and wet deposition of aerosols^68^, FLEXPART includes turbulence^70^, unresolved mesoscale motions^71^, and deep convection^72^. The model output consists of a spatially gridded sensitivity to the respective emissions for the microplastic deposition (wet and dry) for each receptor point^67^. Deposition rates of microplastics (in kg m^− 2^ day^− 1^) can be computed by multiplying the emission sensitivities (in m) with gridded emissions (in kg m^− 2^ day^− 1^), divided by the lowest model layer (100 m). Here, we used emissions from an updated inventory based on Evangeliou et al.^25^. The latter includes sectoral emissions from sea-spray, agricultural nets, resuspension from bare soils, road dust and fibers.

## Supplementary Information

Below is the link to the electronic supplementary material.

Supplementary Material 1

## Data Availability

All data, including all stages of data analysis and processing, can be accessed through the Zenodo Repository under: https://doi.org/10.5281/zenodo.17942873 . Raw mass spectra files will be made available upon request. All FLEXPART results are openly accessible from: https://atmo-access.nilu.no/MPS_HLR2.py.

## Code availability

The workflow code and fingerprinting algorithm are available through the Zenodo Repository under: https://doi.org/10.5281/zenodo.17942873 .
