# Supplementary material for: Nanoplastic detection and quantification in high-altitude glacial snow samples from Italy, Switzerland and France
Source: Sci Rep. 2026 Aug 10;16:24714. doi: 10.1038/s41598-026-63263-y (PMC13457595; doi:10.1038/s41598-026-63263-y)
Supplement: Supplementary file 1 [file 41598_2026_63263_MOESM1_ESM.docx]

**Supplementary Data to**

*Nanoplastic Detection and Quantification in High-Altitude Glacial Snow Samples from Italy, Switzerland and France*

*Milena Latz^1^, Alasdair J. Gill^2^, Nikolaos Evangeliou^3^, Sabine Eckhardt^3^, Robin Milner^2^, Thorsten Reemtsma^1^, Dušan Materić^1^*

*^1^Department of Environmental Analytical Chemistry, Helmholtz Centre for Environmental Research (UFZ), Leipzig, Germany*

*^2^www.high-level-route.com*

*^3^Stiftelsen NILU (former Norwegian Institute for Air Research), Department for Atmospheric & Climate Research (ATMOS), 2007 Kjeller, Norway*

# 1. Supplementary Methods: SOP Sampling Process

Both team members are to remain roped up throughout the sampling process. The rope leader is primarily responsible for safety during the sampling process, whilst the second mountaineer (Nº2) must carry out sampling, recording and record-keeping.

1.	When nearing a sample site, Nº2 prompts Leader to find a safe place to stop (avoiding obvious crevasses, rock fall or other hazards).

2.	Leader decides when to stop the rope team, in collaboration with Nº2 (who will have a better view of the actual sampling site).

3.	Leader remains constantly alert to possible crevasse falls, rock-fall or other hazards.

4.	Nº2 extracts sampling kit (four vials plus HPLC water, notebook, camera, all in cotton bag) before moving to the actual sampling point, which shall be undisturbed snow, at least 5 meters from any obvious tracks.

5.	Nº2 sets up camera on ice axe, with wide angle view of sampling site and starts video recording (optional, depending on conditions).

6.	Nº2 dons gloves (new, nitrile), proceeds to take 3 samples from points forming a triangle with sides of between 1.5 and 3.0 meters.

7.	Nº2 creates a “field blank” by part-filling a fourth vial with HPLC water from the bottle.

8.	Nº2 calls out the reference codes of the vials, time, date, GPS position (from 2 devices) and atmospheric pressure (uncorrected for altitude, from 2 devices) and Leader notes down data.

9.	Nº2 wraps the vials with aluminum foil and packs the used sample kit in their pack before making a final check that no equipment has been left, then returns to the normal track as defined by Leader.

# 2. Sampling Information

Supplementary Table S1.Locational information for sampling sites including description of location, GPS information (latitude, longitude), pressure in hPa, slope in °, fall-line in °, altitude in m, and temperature in °C during at sampling time. Sample jars from sites portrayed in red were leaking during transport and could therefore not be analyzed. The sample from “Italian Peak” of the Matterhorn (in red) could not be clearly assigned to a Site No. and was therefore excluded. FB – Field Blank.

| Site | Location Description | Latitude | Longitude | Pressure  [hPa] | Slope  [°] | Fall line  [°] | Altitude  [m] | Temp  [°C] |
| --- | --- | --- | --- | --- | --- | --- | --- | --- |
| 1 | Schönbielgletscher | 46.02216 | 7.624108 | 700.6 | 12 | 130 |  | 6 |
| X | Schönbielgletscher | 46.01431 | 7.624619 | 708.95 | 4 | 266 |  | 12 |
| 2 | Top of Glacier de Pièce (Col des Vignettes) | 45.99052 | 7.474581 | 700.85 | 31 | 344 | 3119 | 8 |
| 3 | Southern side of Col des Vignettes | 45.98834 | 7.471951 | 695.95 | 10 | 92 | 3170 | 10 |
| 4 | Above Col de Charmontane, top of Otemma | 45.98353 | 7.472674 | 708.45 | 10 | 108 | 3037 | 9 |
| 5 | Snow patch below Pointes des Essettes | 45.95 | 7.065482 | 729.85 | 30 | 225 |  |  |
| 6 | Glacier d'Orny, above the Orny hut | 45.99809 | 7.052308 | 724.95 | 24 | 56 | 2895 | 10 |
| 7 | Trient Plateau | 45.99267 | 7.039663 | 700.9 | 11 | 25 | 3170 |  |
| 8 | 40m from Roc des Plines | 45.98888 | 7.043075 | 694.55 | 9 | 330 | 3245 | 6 |
| X | Snow patch below Trient hut | 45.99822 | 7.042439 | 708.55 | 7 | 224 | 3084 | 14 |
| X | Gl. de Trient, 50m from Fenêtre de Saleinaz | 45.98386 | 7.025049 | 693.85 | 14 | 12 | 3246 | -1 |
| 9 | Top of Saleinaz Glacier, below Fenêtre | 45.98146 | 7.026075 | 701.45 | 17 | 90 | 3171 | 10 |
| 10 | Snow patch below Saleinaz hut | 45.97586 | 7.071221 | 748.35 | 22 | 340 | 2644 | 20 |
| 11 | Snow patch above Leschaux hut | 45.89671 | 6.983175 | 751.55 | 38 | 242 | 2626 | 15 |
| 12 | Snow patch at top of Mer de Glace | 45.88015 | 6.936911 | 773.15 | 14 | 343 | 2364 | 4 |
| 13 | Below the Midi station, 50m from bergschrund | 45.87856 | 6.890646 | 666 | 29 | 140 | 3638 |  |
| 14 | Overlooking Gros Rognon | 45.87685 | 6.891411 | 669 | 8 | 182 | 3613 | 18 |
| 15 | Col du Midi | 45.86578 | 6.895469 | 672.65 | 11 | 10 | 3510 | 16 |
| 16 | SW of Cosmique hut | 45.87037 | 6.885786 | 673.3 | 5 | 310 | 3575 | 18 |
| 17 | Snow slope between Cosmique & Simond huts | 45.874 | 6.88761 | 672.85 | 22 | 104 | 3589 | 20 |
| 18 | Snow platform on Midi Arêt | 45.88024 | 6.889279 | 670.35 |  |  |  |  |
| 19 | Snow patch at top of Cosmique ridge, from Midi station | 45.87808 | 6.887038 | 661.75 | 10 | 0 | 3734 | 18 |
| X | "Italian Peak" of the Matterhorn | 45.97663 | 7.657586 | 652 |  | 144 | 3801 | 16 |
| FB | Schönbiel hut | 46.00192 | 7.628944 |  |  |  |  |  |
| FB | Above Col de Charmontane, top of Otemma | 45.98353 | 7.472674 | 708.45 | 10 | 108 | 3037 | 9 |
| FB | Snow patch below Trient hut | 45.99822 | 7.042439 | 708.55 | 7 | 224 | 3084 | 14 |
| FB | Snow patch below Trient hut | 45.99819 | 7.042444 |  |  |  |  |  |
| FB | Terrace of Trient hut |  |  |  |  |  |  |  |
| FB | Outside the Saleinaz hut |  |  |  |  |  |  |  |
| FB | Outside the Saleinaz hut |  |  |  |  |  |  |  |
| FB | Below Cosmique ridge | 45.8761 | 6.888732 | 670.35 |  |  |  |  |

# 3. Sample Results

Supplementary Table S2. Detected nanoplastic (< 1µm) concentrations in ng mL^-1^±σ. Shown concentrations are Field Blank (FB) corrected. Samples were measured randomized and in triplicates. σ accounts for the standard deviation between triplicate sample measurements. PE – Polyester, PET – Polyethylene, PP – Polypropylene, PS – Polystyrene, PVC – Polyvinylchloride, TWP – Tire Wear Particles.

| Site | PE | PET | PP | PS | PVC | TWP | Total |
| --- | --- | --- | --- | --- | --- | --- | --- |
| 1 | 0 | 0 | 0 | 0 | 0 | 0 | 0 |
| 2 | 41.92±39.90 | 0 | 23.92± 32.99 | 0 | 6.273±15.29 | 0 | 72.11±53.98 |
| 3 | 48.50±46.80 | 0 | 27.83±38.33 | 0 | 0 | 0 | 76.33±60.50 |
| 4 | 4.242±219.09 | 0 | 0.5825±23.26 | 0 | 0 | 0 | 4.824±30.09 |
| 5 | 55.65±10.76 | 0 | 29.36±10.76 | 0 | 0 | 44.53±10.76 | 129.5±18.64 |
| 6 | 0 | 0 | 0 | 0 | 3.627±12.21 | 0 | 3.627±12.21 |
| 7 | 0 | 0 | 0 | 0 | 0 | 0 | 0 |
| 8 | 19.36±38.86 | 0 | 18.22±29.77 | 0 | 0 | 0 | 37.58±48.95 |
| 9 | 5.027±20.71 | 0 | 0.5075±22.10 | 0 | 4.113±12.55 | 0 | 9.648±32.78 |
| 10 | 0 | 0 | 0 | 0 | 9.230±13.44 | 0 | 9.230±13.44 |
| 11 | 52.13±78.56 | 0 | 70.91±76.80 | 6.802±13.81 | 17.08±18.87 | 76.57±60.57 | 223.5±127.83 |
| 12 | 207.0±23.39 | 62.88±22.21 | 125.6±14.41 | 0 | 0 | 74.65±67.37 | 470.1±76.01 |
| 13 | 46.09±53.23 | 0 | 40.12±44.25 | 8.870±13.31 | 18.88±16.39 | 5.381±19.40 | 119.3±74.92 |
| 14 | 0 | 0 | 0 | 7.360±12.74 | 17.94±12.05 | 16.92±18.73 | 42.22±25.65 |
| 15 | 0 | 0 | 0 | 0 | 12.60±11.00 | 0 | 12.60±11.00 |
| 16 | 43.68±48.00 | 0 | 43.28±31.78 | 0 | 10.16±14.15 | 0 | 97.12±59.28 |
| 17 | 0 | 0 | 5.210±31.57 | 0 | 11.78±14.84 | 0 | 16.99±34.88 |
| 18 | 0 | 0 | 0 | 0 | 9.127±13.89 | 0 | 9.127±13.80 |
| 19 | 111.5±16.60 | 23.96±23.40 | 59.35±14.62 | 18.22±19.20 | 0 | 61.86±14.38 | 274.9±40.15 |

# 4. Quality Control

## 4.1 Blanks

Supplementary Table S3. Detected nanoplastic (< 1µm) concentrations in ng mL^-1^±σ. Shown concentrations are Field Blank (FB) corrected. Procedural Blanks (PBs) were taken each day of sample measurement using HPLC water. FBs were taken in the field using HPLC water. Samples were measured randomized and in triplicates. σ accounts for the standard deviation between triplicate measurements. PE – Polyester, PET – Polyethylene, PP – Polypropylene, PS – Polystyrene, PVC – Polyvinylchloride, TWP – Tire Wear Particles.

| Sample | PE | PET | PP | PS | PVC | TWP |
| --- | --- | --- | --- | --- | --- | --- |
| PB_HLR1 | 0 | 0 | 0 | 0 | 0 | 0 |
| PB_HLR2 | 0 | 0 | 0 | 0 | 0 | 0 |
| PB_HLR3 | 0 | 0 | 0 | 0 | 0 | 0 |
| PB_HLR4 | 0 | 0 | 0 | 0 | 0 | 0 |
| PB_HLR5 | 0 | 0 | 0 | 0 | 0 | 0 |
| PB_Mean | 0 | 0 | 0 | 0 | 0 | 0 |
| FB_10 | 0 | 0 | 7.353±12.74 | 0 | 0 | 0 |
| FB_11 | 0 | 0 | 0 | 0 | 0 | 0 |
| FB_42 | 0 | 0 | 0 | 0 | 0 | 0 |
| FB_43 | 0 | 0 | 0 | 0 | 0 | 0 |
| FB_56 | 0 | 0 | 0 | 0 | 0 | 0 |
| FB_58 | 0 | 0 | 18.48±32.01 | 0 | 0 | 0 |
| FB_60 | 0 | 0 | 26.83±23.34 | 0 | 0 | 0 |
| FB_9 | 17.57±30.44 | 0 | 42.79±5.839 | 0 | 0 | 0 |
| FB_Mean | 2.197±10.76 | 0 | 11.93±19.73 | 0 | 0 | 0 |

## 4.2 Spiking Results

Supplementary Table S4. Calculated mass concentrations in ng for samples spiked with 100 ng polystyrene (PS) solution. Samples were measured randomized and in triplicates. PB – Procedural Blank

| Sample | PS  [ng] | σ  [ng] | Recovery  [%] |
| --- | --- | --- | --- |
| 10_PS | 90.27 | 14.64 | 90 |
| 15_PS | 118.1 | 18.36 | 118 |
| 1_PS | 86.47 | 16.33 | 86 |
| 58_PS | 97.95 | 14.62 | 98 |
| 78_PS | 116.3 | 16.19 | 116% |
| 9_PS | 103.4 | 10.15 | 103% |
| Mean_PS | 102.08 | 15.05 | 102% |
| PB_2 | 0 | 0 | - |

# 5. Plastic Library

Supplementary Table S5. Top 40 qualifying ions used during fingerprinting for six common polymer types. PE – Polyester, PET – Polyethylene, PP – Polypropylene, PS – Polystyrene, PVC – Polyvinylchloride, TWP – Tire Wear Particles.

| **Rank [Int]** | **PET [*m/z*]** | **PP [*m/z*]** | **PE [*m/z*]** | **PS [*m/z*]** | **PVC [*m/z*]** | **TWP [*m/z*]** |
| --- | --- | --- | --- | --- | --- | --- |
| 1 | 149.03 | 101.059 | 101.059 | 105.065 | 107.049 | 107.048 |
| 2 | 123.044 | 123.113 | 121.064 | 107.049 | 129.089 | 123.042 |
| 3 | 181.083 | 109.099 | 113.057 | 121.064 | 149.03 | 101.058 |
| 4 | 105.034 | 113.057 | 115.075 | 123.044 | 121.064 | 111.046 |
| 5 | 151.032 | 111.075 | 125.058 | 117.087 | 123.044 | 109.097 |
| 6 | 167.034 | 195.154 | 111.075 | 106.072 | 257.246 | 105.033 |
| 7 | 121.064 | 125.094 | 129.089 | 133.064 | 131.084 | 121.098 |
| 8 | 150.036 | 115.075 | 123.044 | 108.053 | 133.064 | 113.058 |
| 9 | 193.049 | 121.1 | 131.084 | 103.041 | 143.084 | 135.041 |
| 10 | 124.047 | 207.109 | 149.03 | 209.096 | 105.065 | 133.059 |
| 11 | 175.039 | 141.089 | 153.093 | 207.109 | 155.08 | 139.078 |
| 12 | 165.09 | 139.106 | 207.109 | 149.03 | 181.1 | 125.093 |
| 13 | 131.084 | 153.093 | 195.154 | 119.083 | 135.046 | 119.079 |
| 14 | 195.119 | 129.089 | 109.099 | 131.104 | 101.024 | 127.074 |
| 15 | 139.04 | 167.138 | 141.089 | 145.105 | 130.073 | 108.053 |
| 16 | 137.06 | 107.082 | 127.075 | 118.063 | 108.053 | 137.058 |
| 17 | 106.036 | 137.122 | 139.077 | 223.166 | 157.094 | 115.074 |
| 18 | 257.246 | 127.105 | 137.06 | 104.076 | 183.082 | 307.128 |
| 19 | 125.024 | 151.138 | 103.073 | 135.046 | 117.087 | 157.091 |
| 20 | 191.094 | 149.123 | 165.09 | 124.047 | 119.083 | 153.088 |
| 21 | 147.043 | 165.146 | 107.049 | 129.089 | 167.083 | 124.045 |
| 22 | 163.043 | 223.166 | 117.087 | 221.108 | 285.277 | 147.076 |
| 23 | 133.031 | 135.079 | 171.139 | 143.084 | 147.043 | 151.08 |
| 24 | 179.105 | 163.14 | 143.105 | 109.029 | 215.084 | 141.088 |
| 25 | 119.048 | 143.105 | 181.123 | 219.114 | 199.077 | 129.089 |
| 26 | 285.277 | 102.064 | 155.104 | 115.075 | 115.018 | 167.091 |
| 27 | 177.056 | 155.104 | 135.079 | 181.123 | 173.124 | 117.056 |
| 28 | 173.124 | 181.123 | 157.12 | 237.08 | 179.065 | 122.037 |
| 29 | 199.077 | 197.15 | 203.086 | 134.067 | 150.036 | 100.041 |
| 30 | 122.034 | 183.137 | 199.17 | 211.093 | 141.056 | 161.059 |
| 31 | 168.036 | 179.132 | 167.138 | 205.098 | 113.024 | 131.069 |
| 32 | 176.044 | 124.119 | 151.083 | 195.154 | 159.044 | 145.089 |
| 33 | 109.029 | 311.296 | 169.119 | 235.067 | 109.029 | 143.073 |
| 34 | 148.044 | 193.121 | 201.099 | 233.121 | 161.063 | 159.107 |
| 35 | 152.064 | 191.164 | 183.137 | 122.061 | 128.044 | 155.103 |
| 36 | 194.051 | 209.152 | 185.155 | 183.082 | 169.096 | 106.038 |
| 37 | 219.049 | 177.135 | 197.15 | 147.081 | 258.248 | 207.106 |
| 38 | 107.049 | 227.203 | 215.084 | 193.098 | 111.045 | 195.117 |
| 39 | 141.056 | 279.231 | 147.043 | 132.086 | 125.024 | 185.141 |
| 40 | 182.057 | 225.184 | 209.152 | 249.076 | 124.047 | 110.103 |

# 6. Statistical Analysis

## 6.1. Pearson Correlation

Figure S1. Pearson Correlation of total plastic concentration [ng/m^3^] measured per site and site-specific parameters: a) slope [°] b) fall line [m] and c) altitude [m].

## 6.2. Principal Component Analysis (PCA)

In the following, we have included three exploratory PCA. For our 19 observations (sampling sites), we have specified 9 continuous variables: concentration values for the six detected polymer types as well as 3 site specific parameters (slope, altitude and fall line). To avoid redundancy in the dataset, we have excluded local pressure as a variable due to their direct correlation to altitude (Pearson correlation r=-0.99) from the PCA. Three of the observations did not specify any values for altitude, we have thus estimated their elevation using the Elevation Finder from FreeMapTools (https://www.freemaptools.com/elevation-finder.htm).

Figure S2. Principal Component Analysis (PCA) of the presented dataset based on: a) all 9 continuous variables, b) only concentration variables for six polymer types (PE, PP, PS, PVC, PET, TWP), c) only site-specific parameters (fall line, slope, altitude).

## 6.3. K-means Clustering

Figure 3. K-means Cluster Plot (K=3). Formation of three distinct clusters based on the site’s altitude: Cluster 1 – high altitude sites (≥3575 m), Cluster 2 – medium altitude sites (3037-3510 m), Cluster 3 – low altitude sites (≤2895 m). Individuals represent sampling sites. Rows including missing data were omitted from the analysis.

# 7. Supplementary Equations: Upscaling

## Conversion from ng mL^-1^ to kg km^-2^

$$A_{sampled}=r_{jar}^{2}* \pi= (2.5 c{m)}^{2}* \pi=15.7 cm^{2}$$

(S1)

$$r_{conversion}=\frac{A_{sampled}}{V_{snowcollected}}=\frac{15.7 cm^{2}}{41 mL}=0.38 cm^{2}mL^{-1}$$

(S2)

$$c_{NPs}=85 ng mL^{-1}*r_{conversion}=\frac{85 ng mL^{-1}}{0.38 cm^{2}mL^{-1}}*0.01=2.2 kg{km}^{-2}$$

(S3)

With:
A_sampled_ – sampled area of surface snow
r_conversion_ – conversion rate
V_snowcollected_ – mean volume of collected snow per jar
c_NPs_ – mean mass concentration of NPs detected

## Upscaling to alpine area

$$A_{alpine2016}=1806\pm60 km^{2}$$

(S4)

$$A_{alpine2022}={A_{alpine2016}-(A}_{alpine2016}*r_{Shrinkage}*(2022-2016))=1806 km^{2}-(1806 km^{2}*0.012 a^{-1}*6 a)=1676\pm56 km^{2}$$

(S5)

$$A_{acc2022}=A_{alpine2022}*AAR=1676 km^{2}*0.58=972\pm322 km^{2}$$

(S6)

$$c_{NPsalpine}= c_{NPs}*A_{alpine}=2.2 kg km^{-2}*1102 km^{2}=2.4\pm1.5 t$$

(S7)

With:
A_alpine2016_ – glacial surface in the European Alps in 2016 according to Paul et al.^1^
A_alpine2022_ – estimated glacial surface in the European Alps in 2022
r_shrinkage_ – glacial shrinkage factor per year according to Paul et al.^1^
A_acc2022_ – estimated glacial accumulation zone in the European Alps in 2022
AAR – global accumulation area ratio according to Oien et al.^2^
c_NPsalpine_ – estimated mean concentration of NPs in the European Alps

## Error propagation

$$\delta_{1}= \sqrt{\left( \frac{A_{alpine2022}}{\sigma_{alpine2022}} \right)^{2}+\left( \frac{AAR}{\sigma_{AAR}} \right)^{2}}= \sqrt{\left( \frac{1676 km^{2}}{56 km^{2}} \right)^{2}+\left( \frac{0.58}{0.19} \right)^{2}}= 0.33$$

(S8)

$${RSE}_{3}=\frac{\sigma}{\sqrt{n}* \bar{X}}=\frac{3.17 kg km^{-2}}{\sqrt{19}*2.21 kg km^{-2}}= 0.33$$

(S9)

$$\delta_{4}=\frac{\Delta X}{X}=\frac{10 mL}{41 mL}=0.24$$

(S10)

$${RSE}_{total}= \sqrt{\delta_{1}^{2}+ \delta_{2}^{2}+ {RSE}_{3}^{2}+ \delta_{4}^{2})}= \sqrt{{0.33}^{2}+{0.30}^{2}+{0.33}^{2}+{0.24}^{2}}=0.61=61\%$$

(S11)

With:
*δ_1_* – relative uncertainty of glacial accumulation area (AAR)
*δ_2_* – systematic error of analytical method used
*RSE_3_* – relative standard error of concentration mean value
δ _4_ – relative uncertainty of sampled snow volume
RSE_total_ – total relative standard error

# 8. References

1. Paul, F. *et al.* Glacier shrinkage in the Alps continues unabated as revealed by a new glacier inventory from Sentinel-2. *Earth Syst. Sci. Data* **12,** 1805–1821; 10.5194/essd-12-1805-2020 (2020).
2. Oien, R. P., Rea, B. R., Spagnolo, M., Barr, I. D. & Bingham, R. G. Testing the area–altitude balance ratio (AABR) and accumulation–area ratio (AAR) methods of calculating glacier equilibrium-line altitudes. *J. Glaciol.* **68,** 357–368; 10.1017/jog.2021.100 (2022).
